# Genetic Steps to Organ Laterality in Zebrafish

**DOI:** 10.1002/cfg.74

**Published:** 2001-04

**Authors:** Jau-Nian Chen, Frauke van Bebber, Allan M. Goldstein, Fabrizio C. Serluca, Donald Jackson, Sarah Childs, George Serbedzija, Kerri S. Warren, John D. Mably, Per Lindahl, Alan Mayer, Pascal Haffter, Mark C. Fishman

**Affiliations:** 1 Cardiovascular Research Center Massachusetts General Hospital 149 13th Street Charlestown MA 02129 USA; 2 Department of Medicine Harvard Medical School Boston MA 02115 USA; 3 Max-Planck-Institut für Entwicklungsbiologie Abteilung Genetik Spemannstrasse 35 Tübingen 72076 Germany

## Abstract

All internal organs are asymmetric along the left–right axis. Here we report a genetic screen to discover mutations which perturb organ laterality. Our particular focus is upon whether, and how, organs are linked to each other as they achieve their laterally asymmetric positions. We generated mutations by ENU mutagenesis and examined F3 progeny using a cocktail of probes that reveal early primordia of heart, gut, liver and pancreas. From the 750 genomes examined, we isolated seven recessive mutations which affect the earliest left–right positioning of one or all of the organs. None of these mutations caused discernable defects elsewhere in the embryo at the stages examined. This is in contrast to those mutations we reported previously (Chen *et al.*, 1997) which, along with left–right abnormalities, cause marked perturbation in gastrulation, body form or midline structures. We find that the mutations can be classified on the basis of whether they perturb relationships among organ laterality. In Class 1 mutations, none of the organs manifest any left–right asymmetry. The heart does not jog to the left and normally leftpredominant *BMP4* in the early heart tube remains symmetric. The gut tends to remain midline. There frequently is a remarkable bilateral duplication of liver and pancreas. Embryos with Class 2 mutations have organotypic asymmetry but, in any given embryo, organ positions can be normal, reversed or randomized. Class 3 reveals a hitherto unsuspected gene that selectively affects laterality of heart. We find that visceral organ positions are predicted by the direction of the preceding cardiac jog. We interpret this as suggesting that normally there is linkage between cardiac and visceral organ laterality. Class 1 mutations, we suggest, effectively remove the global laterality signals, with the consequence that organ positions are effectively symmetrical. Embryos with Class 2 mutations do manifest linkage among organs, but it may be reversed, suggesting that the global signals may be present but incorrectly orientated in some of the embryos. That laterality decisions of organs may be independently perturbed, as in the Class 3 mutation, indicates that there are distinctive pathways for reception and organotypic interpretation of the global signals.


**Comparative and Functional Genomics**
*Comp Funct Genom* 2001; **2**: 60–68.
**DOI**: 10.1002/cfg.74

**Research Article**

### Genetic steps to organ laterality in zebrafish

Jau-Nian Chen[1], Frauke van Bebber[2], Allan M. Goldstein[1], Fabrizio C. Serluca[1], Donald Jackson[1], Sarah Childs[1], George Serbedzija[1], Kerri S. Warren[1], John D. Mably[1], Per Lindahl[1], Alan Mayer[1], Pascal Haffter[2] and Mark C. Fishman[1]*

[1] *Cardiovascular Research Center, Massachusetts General Hospital, 149 13th Street, Charlestown, MA 02129, and Department of Medicine, Harvard Medical School, Boston, MA 02115, USA*
[2] *Max-Planck-Institut für Entwicklungsbiologie, Abteilung Genetik, Spemannstrasse 35, 72076, Tübingen, Germany*

*Correspondence to:
M. C. Fishman, Cardiovascular Research Center, Massachusetts General Hospital, 149 13th Street, Charlestown, MA 02129, USA and Department of Medicine, Harvard Medical School, Boston, MA 02115, USA.
E-mail:
fishman@cvrc.mgh.harvard.edu*

Received: 29 November 2000
Accepted: 23 February 2001

### Abstract

**All internal organs are asymmetric along the left–right axis. Here we report a genetic screen to discover mutations which perturb organ laterality. Our particular focus is upon whether, and how, organs are linked to each other as they achieve their laterally asymmetric positions. We generated mutations by ENU mutagenesis and examined F3 progeny using a cocktail of probes that reveal early primordia of heart, gut, liver and pancreas. From the 750 genomes examined, we isolated seven recessive mutations which affect the earliest left–right positioning of one or all of the organs. None of these mutations caused discernable defects elsewhere in the embryo at the stages examined. This is in contrast to those mutations we reported previously (Chen *et al.*, 1997) which, along with left–right abnormalities, cause marked perturbation in gastrulation, body form or midline structures. We find that the mutations can be classified on the basis of whether they perturb relationships among organ laterality. In Class 1 mutations, none of the organs manifest any left–right asymmetry. The heart does not jog to the left and normally left-predominant *BMP4* in the early heart tube remains symmetric. The gut tends to remain midline. There frequently is a remarkable bilateral duplication of liver and pancreas. Embryos with Class 2 mutations have organotypic asymmetry but, in any given embryo, organ positions can be normal, reversed or randomized. Class 3 reveals a hitherto unsuspected gene that selectively affects laterality of heart. We find that visceral organ positions are predicted by the direction of the preceding cardiac jog. We interpret this as suggesting that normally there is linkage between cardiac and visceral organ laterality. Class 1 mutations, we suggest, effectively remove the global laterality signals, with the consequence that organ positions are effectively symmetrical. Embryos with Class 2 mutations do manifest linkage among organs, but it may be reversed, suggesting that the global signals may be present but incorrectly orientated in some of the embryos. That laterality decisions of organs may be independently perturbed, as in the Class 3 mutation, indicates that there are distinctive pathways for reception and organotypic interpretation of the global signals. Copyright © 2001 John Wiley & Sons, Ltd.**

**Keywords:** **symmetry; heterotaxy; *situs inversus*; fork head gene**

### Introduction

The break of embryonic left–right symmetry initiates cascades of asymmetrically expressed genes and eventually places internal organs asymmetrically in vertebrates. How the embryonic symmetry breaks is not yet clear, though cortical rotation and gap junction channels have been suggested to play a role in *Xenopus* and chick embryos (Levin and Mercola, 1998). By early gastrulation, there are higher levels of *shh* expression on the left side of the node in chick embryos (Levin *et al.*, 1995). This asymmetry

Copyright © 2001 John Wiley & Sons, Ltd.

then induces left-sided expression of *nodal*, *lefty 1*, *lefty 2*, *pitx2* (for review, see Ramsdell and Yost, 1998) and *caronte* (Rodriguez Esteban *et al.*, 1999; Yokouchi *et al.*, 1999). Experimentally rendering these gene activities symmetric by overexpression leads to symmetric expression of downstream genes (for review, see Ramsdell and Yost, 1998). The consequence of such signal homogenization is a 'randomization' of organ laterality, in which there is an unpredictable outcome from embryo to embryo and between organs (Schilling *et al.*, 1999; Ramsdell and Yost, 1998). There is neither normal nor reversed *situs* in injected embryos. Hence, organs become 'unlinked' from each other.

Loss-of-function mutations have a different effect than do embryos overexpressing normally asymmetric genes. For example, despite a certain degree of heterotaxy, about 50% of homozygous *iv –/–* (defective in *left/right dynein* (Supp *et al.*, 1997) progeny have *situs inversus* and 50% *situs solitus* (Hummel and Chapman, 1959). This same pattern of inheritance is noted in human patients with Kartagener syndrome. It is hard to explain how normal and reversed *situs* occur in different progeny with the same genetic deficiency. These observations suggest that in these disorders there is retained linkage among organs.

To better understand the genetic nature of asymmetry linkage among organs, we sought mutations that perturb cardiac laterality in the zebrafish. As one group, we examined mutations previously discovered by dint of other phenotypes. We previously found 21 mutations that perturbed the heart's asymmetry, all of which also had pronounced midline or gastrulation defects (Chen *et al.*, 1997). These fall into two groups: one class of mutation abrogates the first evidence of cardiac asymmetry (left-predominant *BMP4* in the early heart tube) and prevents the occurrence of the first morphological asymmetry, the jog of the heart tube to the left. Embryos with mutations of the other class may have either a normal or reversed pattern of *BMP4* in the heart tube, which predicts normal or reversed cardiac looping, respectively. All the laterality mutants isolated from this prior screen bear additional defects, either in dorsoventral patterning or in midline structures (Chen *et al.*, 1997). This is in agreement with the notion that proper dorsoventral patterning and midline structures have profound influence on the left–right axis (Danos and Yost, 1995; Danos and Yost, 1996).

In addition, we undertook a new genetic screen seeking mutations that specifically perturb laterality of the earliest organ primordia. We found seven mutations that disrupt organotypic laterality without evident effect upon midline development or gastrulation. We report here this new set of mutations and additional analysis of the prior group, specifically with regard to linkage of laterality decisions among organ primordia. We find one class of mutations that appear to consistently remove all laterality information in every embryo, causing relative symmetry across the midline. A second class of mutations variably affects organ laterality in different embryos of a single clutch, such that some mutant embryos manifest *situs solitus*, some *situs inversus* and some heterotaxy. In addition, we find a mutation with a selective role in laterality decision made by one individual organ.

### Materials and methods

### Mutagenesis

The mutagenesis and screening was done as described previously (Mullins *et al.*, 1994). Tup longfin (TL) male fish were mutagenized by ENU as described in Mullins *et al.* (1994). In short, the male fish were treated with 3 mM ENU for 1 h/day, every other day, for a total of three treatments. These fish were outcrossed to wild-type TL females 3 weeks after ENU treatment, to generate $F_1$ families. $F_2$ families were generated by random incrossing of the $F_1$ fish. The mutant phenotypes were analysed in the $F_3$ embryos.

### Determination of cardiac *situs*

At 24 hours post-fertilization (hpf) the direction of cardiac jogging was determined as previously described (Chen *et al.*, 1997). Embryos were divided into three groups, left jog (normal), right jog (reversed) or midline (no jog). The midline group designates hearts that fail to jog, remaining within the borders of the overlying neural tube when visualized under a dissecting microscope. PTU (0.2 mM 1-phenyl-2-thiourea) was added at 24 hpf to prevent pigmentation which would obscure later *in situ* hybridization staining. At 48 hpf cardiac looping was analysed and each of the previously described three groups was further subdivided as follows: right-loop (normal), left-loop (reversed), or

Copyright © 2001 John Wiley & Sons, Ltd.

no loop. Each group of embryos was subsequently fixed in 4% paraformaldehyde to be processed for whole-mount *in situ* hybridization.

### Visceral *situs* determination

Whole-mount *in situ* hybridization was performed on 48 hpf embryos as described (Chen and Fishman, 1996) using digoxigenin-labelled antisense RNA probes. *fkd2* was used to analyze the position of liver, and *pdx* for gut and pancreas (Milewski *et al.*, 1998). For the assessment of visceral organ laterality, only *pdx* and *fkd2* proved particularly instructive, and are described here.

### Results

### Screening for laterality mutations

Despite attention to cardiac looping, no laterality mutations were identified during the two large-scale genetic screens in zebrafish. Several factors may have been responsible for this. First, organ formation is a relatively late event during development, and since genes involved in organogenesis may have roles in earlier embryogenesis as well, the early effects of mutations may have obscured later organotypic ones. Second, it is difficult to distinguish organ precursors and early organ primordia without the aid of specific markers. In addition, genetic defects that randomize looping are evident as such in only half of affected embryos, reducing the number of apparently affected progeny. Hence, we subsequently re-evaluated mutants isolated from the original screens using the cardiac jog and a marker (BMP4) expressed asymmetrically within the heart, and thereby found 21 to disrupt cardiac laterality (Chen *et al.*, 1997). All of these mutations had been originally identified because the affected embryos had other prominent phenotypes, usually affecting midline structures or gastrulation. In the current screen reported here, we sought evidence of laterality deficits as the principle, in fact only, phenotype to justify mutation isolation.

In addition to assessing cardiac laterality as before, we used *fkd2* to reveal the liver primordia and *pdx* to highlight gut and pancreas after 2 days of development. The *fkd2* gene is a member of the winged helix family of transcription factors. The early pattern of *fkd2* expression (prior to 24 hpf) has been analysed in detail (Odenthal and Nüsslein-Volhard, 1998) but expression at later stages is not reported. We find that *fkd2* expression is a marker for gut primordia beginning at 24 hpf. After 3 days of development, its expression becomes prominent in the liver and pancreas as well (Figure 1A). *pdx* is a homeodomain gene that marks the developing gut and pancreas (Milewski *et al.*, 1998) (Figure 1B). With these two probes, we are able to visualize the liver and gut primordia on the left and pancreas on the right of the embryo after 2 days of development. From the 750 genomes examined, we isolated seven mutations that have as their only evident defect disruption of organ laterality.

### Linkage between cardiac and visceral organ laterality decisions

Our principle focus here was upon the genes that drive laterality relationships among organs. We assessed this by examining the seven new mutations we isolated in this screen, which have no other obvious defects, and 14 of those we previously reported to disturb laterality in the context of midline or gastrulation defects.

We find that visceral organ position can be predicted by the direction of cardiac jogging. Normally, the heart jogs to the left and later loops to the right. In the abdomen, the liver develops on the left, the pancreas on the right, and the gut moves to the left of the midline. It appears that the mere presence of the cardiac jog, whether normal or reversed, is a powerful predictor of retention of linkages among all organs. In other words, from all mutant embryos (which from the prior screen we can determine by associated phenotypes), if the heart jogs to the left, visceral *situs* is normal. As shown in Table 1, this is true, regardless of the mutation, for all 21 mutations analysed (from a total of 249 embryos, only four had abnormal visceral *situs* with a normal jog). In contrast, a reversed jog (to the right) is consistently associated with totally reversed visceral *situs*. Regardless of the underlying mutation, as shown in Table 1, all of the 185 mutant embryos that manifest a rightward jog display a complete reversal of all organ positions. In these cases, the liver and gut are on the right, the pancreas is on the left, and the heart subsequently loops to the right (Figure 2). We refer to this situation as *situs inversus*. Mutant embryos that do not have a directional jog (C-jog in Table 1; $n = 424$), subsequently manifest no apparent linkage

Copyright © 2001 John Wiley & Sons, Ltd. 

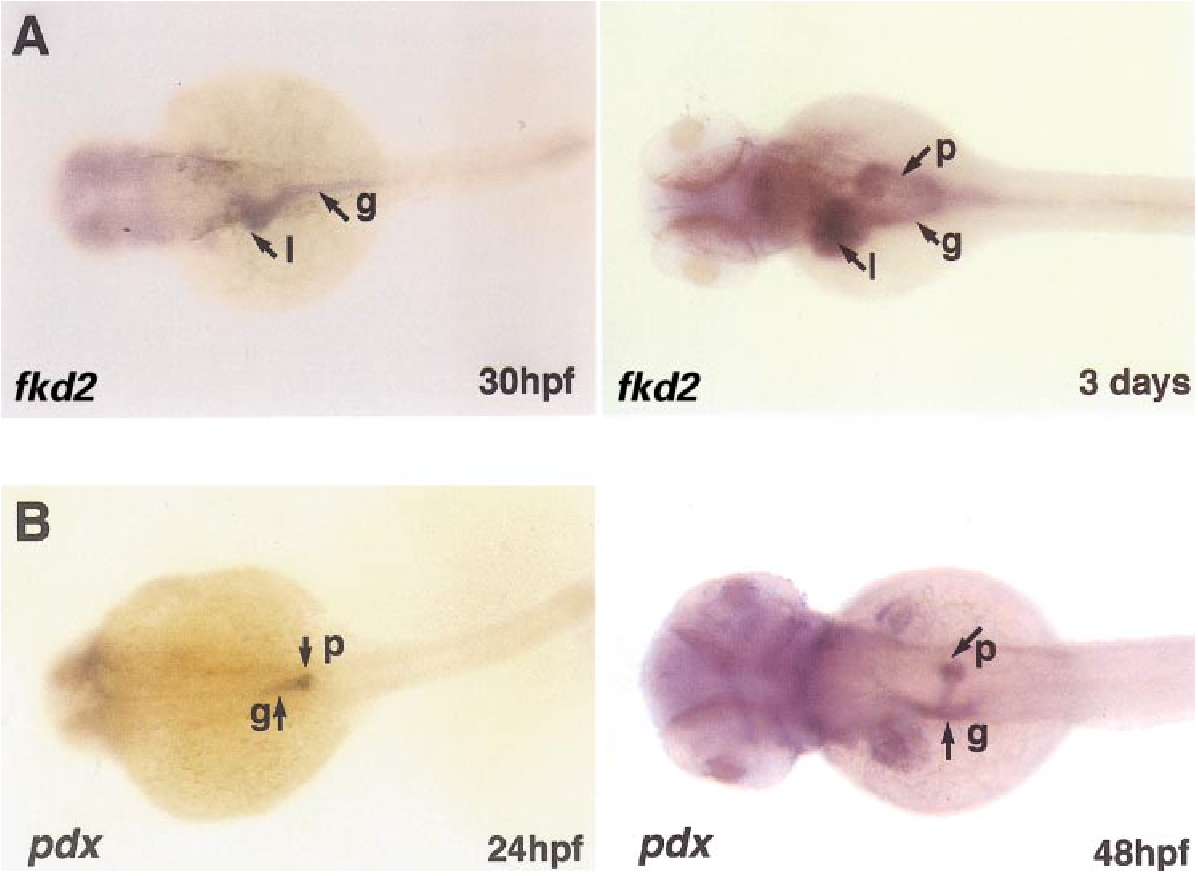

**Figure 1.** Asymmetry of visceral organ primordia revealed by (A) *fkd2* probe and (B) *pdx* probe. Liver (l); gut (g); pancreas (p)

between the direction of cardiac looping and visceral organ *situs*, and are not predictable in orientation. The majority (68%) have discordant organ positions (heterotaxy, see below).

Interestingly, the mutations appeared to fall principally into two classes, one in which most or all embryos lack evident linkages between organotypic laterality (Class 1) and the other (Class 2) in which a significant proportion of mutant embryos retain linkage, manifested either as *situs solitus* or *situs inversus*. In addition, we discovered one mutation that affects laterality of a single organ, a type of defect we refer to as Class 3.

Table 1. Linkage between cardiac and visceral organ laterality

| | Situs solitus | Situs inversus | Heterotaxy |
|---|---|---|---|
| L-jog, *n*=249 | 245 (98.4%) | 2 (0.8%) | 2 (0.8%) |
| C-jog, *n*=424 | 61 (14%) | 75 (18%) | 288 (68%) |
| R-jog, *n*=185 | 0 (0%) | 185 (100%) | 0 (0%) |

*Situs solitus* means that all organs are normally situated. *Situs inversus* means that all organs are mirror-image reversed in left–right orientation. Heterotaxy means that there is a discordance among the laterality of different organs. L-jog, R-jog, C-jog (or 'no jog') are described in the text.

### Class 1 mutations: predominance of heterotaxy

This class includes *ntl*, *flh* and *sur*. As we noted previously (Chen *et al.*, 1997), the heart fails to jog in all embryos of this class. These midline hearts subsequently do bend, but the direction is unpredictable and the mechanism may be distinct from that of normal looping (Chen *et al.*, 1997).

As summarized in Table 2, the vast majority of such embryos also have abnormalities in visceral organ *situs*. There is no predictability or consistency to the arrangement of the organs. Furthermore, *situs inversus* does not occur (except in 1% of *ntl* embryos),

Copyright © 2001 John Wiley & Sons, Ltd. 

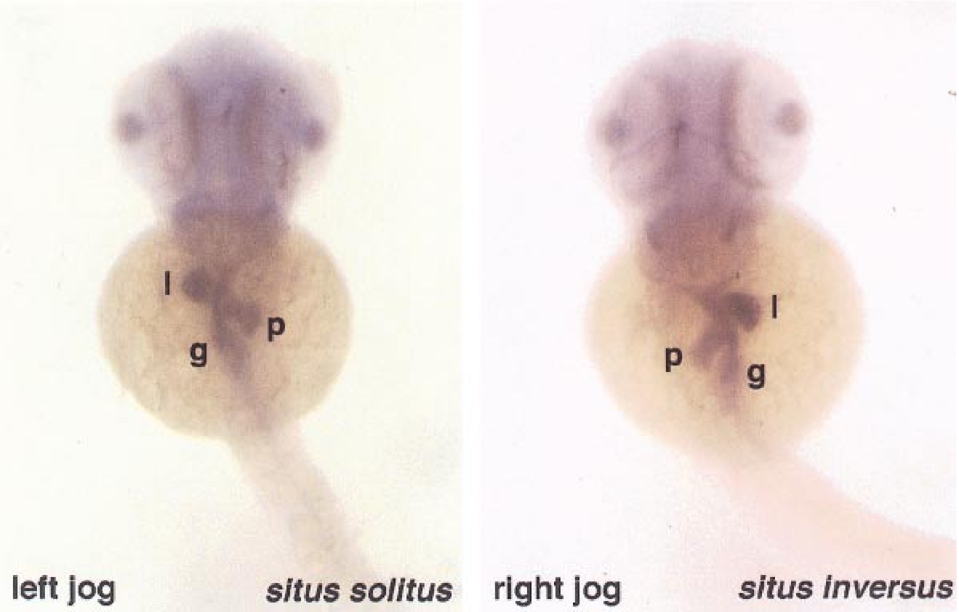

**Figure 2.** Embryos with a left jog (left panel) always exhibit normal *situs*, with gut and liver on the left and pancreas on the right (*situs solitus*). Embryos with a right jog (right panel) have reversed placement of viscera (*situs inversus*)

which is very different from Class 2 mutants (see below). The *sur* mutant embryos appear to have the weakest effect, in that 17% of embryos have a normal arrangement of visceral organs. We do not know if this is a penetrance effect.

In addition, as noted in Table 2, there is frequently a bilateral duplication of the liver, the pancreas, or both. Sometimes this is relatively symmetric, as shown in Figure 3A. In some embryos the right liver is predominant (Figure 3B) and in others it is the left (Figure 3C). To our knowledge, this bilateral duplication of liver or pancreas has not been reported previously as a consequence of mutations. It is conceivable that species differ in which organs are subject to duplication. For example, in humans, *situs* disturbances may be associated with the presence of multiple spleens. Isomerism of the lungs or atria also occurs in human, but this term used clinically does not refer to an additional structure but rather to a loss of distinctiveness between the two sides (Burn, 1991).

### Class 2 mutations: coexistence of *situs solitus*, *situs inversus* and heterotaxy

This class includes 18 mutations, as shown in Table 2. The hallmark of this class is that, unlike Class 1, some mutant embryos do manifest organ laterality and frequently a linkage among the organs. The heart of an individual embryo may jog to the left or jog to the right or remain at the midline (Chen *et al*., 1997). As noted, leftward jog is predictive of *situs solitus* of the liver, pancreas and gut (Figure 4A). A rightward jog is associated with *situs inversus* of all visceral organs (Figure 4B). In the embryos in which the heart does not jog, there is, as in Class 1, a random positioning of organs, and in some cases, bilateral organ duplication (Figure 4C; Table 2).

### Class 3 mutation: Organ-specific laterality defect

Lost-a-fin is one of the five dorsalized mutants isolated from the large-scale genetic screens (Mullins *et al*., 1996). We find that the mutant embryos of *laf* exhibit abnormal *situs* for one organ but not the others. Embryos with the *laf* mutation do not manifest a directional cardiac jog or lateralization of looping, but visceral *situs* is generally normal (98%, $n = 59$) (Table 2). This suggests that *laf* is a component of mechanisms which determine laterality of heart but not viscera.

Copyright © 2001 John Wiley & Sons, Ltd. 

Table 2. Organ laterality of the subset of embryos in which the heart fails to jog

| Mutant | Situs solitus | Situs inversus | Discordant | Duplication |
|---|---|---|---|---|
| Class 1 | | | | |
| sur, $n=24$ | 4 (17%) | 0 (0%) | 15 (63%) | 5 (20%) |
| flh, $n=16$ | 1 (6%) | 0 (0%) | 1 (6%) | 14 (88%) |
| ntl, $n=83$ | 3 (4%) | 1 (1%) | 25 (30%) | 54 (65%) |
| Class 2 | | | | |
| tw29b, $n=16$ | 0 (0%) | 0 (0%) | 10 (62.5%) | 6 (37.5%) |
| tm317b, $n=33$ | 14 (42%) | 6 (18%) | 11 (33%) | 2 (6%) |
| cup, $n=24$ | 1 (4%) | 4 (17%) | 9 (37%) | 10 (42%) |
| n20b, $n=3$ | 0 (0%) | 0 (0%) | 1 (33%) | 2 (66%) |
| pgy, $n=12$ | 1 (8%) | 0 (0%) | 0 (0%) | 11 (92%) |
| spt, $n=11$ | 1 (9%) | 1 (9%) | 8 (72%) | 1 (9%) |
| dino, $n=16$ | 3 (19%) | 0 (0%) | 12 (75%) | 1 (6%) |
| an22, $n=40$ | 0 (0%) | 7 (17.5%) | 29 (72.5%) | 4 (10%) |
| am043, $n=19$ | 2 (11%) | 6 (31%) | 7 (37%) | 4 (21%) |
| AO37, $n=8$ | 2 (25%) | 0 (0%) | 4 (50%) | 2 (25%) |
| AG038, $n=79$ | 11 ($n=14\%$) | 14 (18%) | 32 (40%) | 22 (28%) |
| fv063b, $n=9$ | 1 (11%) | 0 (0%) | 7 (78%) | 1 (11%) |
| fv061b, $n=16$ | 1 (6%) | 1 (6%) | 13 (82%) | 1 (6%) |
| fe07, $n=7$ | 2 (29%) | 1 (14%) | 3 (43%) | 1 (14%) |
| fw01, $n=15$ | 1 (7%) | 3 (20%) | 11 (73%) | 0 (0%) |
| fv33a, $n=6$ | 0 (0%) | 1 (16.5%) | 4 (67%) | 1 (16.5%) |
| fnl2b, $n=7$ | 1 (14%) | 1 (14%) | 5 (72%) | 0 (0%) |
| fs07, $n=9$ | 1 (11%) | 2 (22%) | 6 (67%) | 0 (0%) |
| Class 3 | | | | |
| laf, $n=59$ | 0 (0%) | 0 (0%) | 58 (98%) | 1 (2%) |

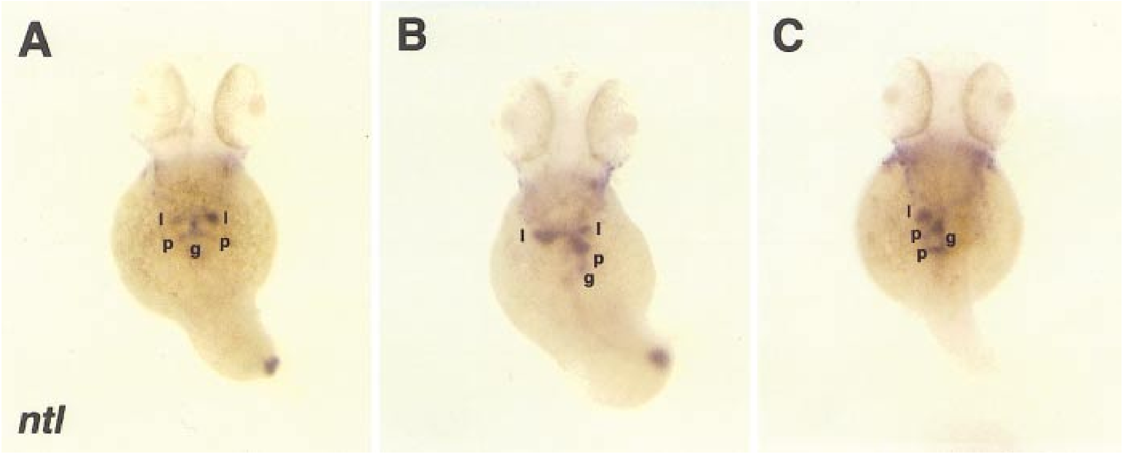

**Figure 3.** Bilateral duplication of visceral organs in Class 1 mutant embryos. Dorsal views of *ntl* embryos at 48 h post-fertilization following *in situ* hybridization with *fkd2* and *pdx* riboprobes, showing a sampling of visceral laterality phenotypes within a single cross seen in Class 1 mutants. (A) Bilateral isomerism of liver (l) and pancreas (p), with a midline gut (g). (B) Left-sided gut, right-sided pancreas, and a bilateral liver, more prominent on the left. (C) Midline gut, left-sided liver, and two left-sided pancreatic primordia

Copyright © 2001 John Wiley & Sons, Ltd. 

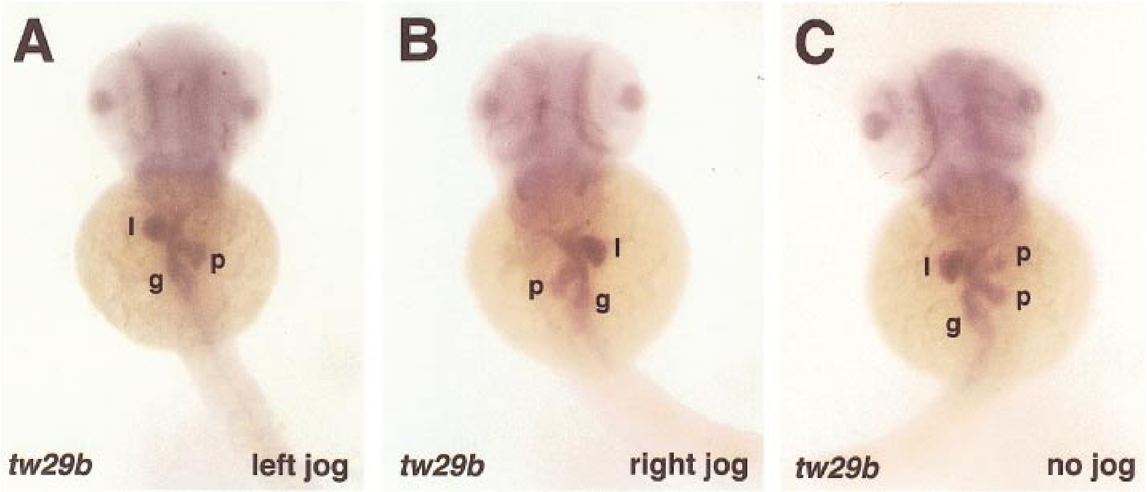

**Figure 4.** Linkage among visceral organs in Class 2 mutant embryos. Dorsal views of *tw29b* mutant embryos stained for *fkd2* and *pdx* expression, demonstrating the abnormalities of *situs* within a single cross in Class 2 mutants. (A) and (B) are examples of embryos with visceral *situs solitus* and *situs inversus*, respectively. In (C), an embryo is which the heart manifested no jog, there appear two right-sided pancreatic buds. Liver (l); gut (g); pancreas (p)

### Discussion

We focus here upon the nature of organotypic asymmetry. In particular, we are interested in whether organs, separated spatially throughout the embryo, utilize the same or different laterality information, so that, under normal circumstances, all organs come to be correctly placed and orientated along the left–right axis.

We describe the linkage among left–right laterality decisions made by four organs, and their perturbation in 21 mutations. Seven we isolated in the screen reported here, which was specifically designed to evaluate asymmetry phenotypes. The seven mutations isolated from this recent screen are different from those described previously in that laterality decisions are the primary defect evident. No significant dorsoventral or midline abnormalities are noted in these mutant embryos, suggesting these genes may act downstream or in parallel to those that determine early embryonic axial patterning.

We find that the first asymmetric movement of the heart (the 'jog') predicts linkage among organs: a normal jog predicts *situs solitus* of liver, pancreas and gut; a reversed jog predicts *situs inversus totalis*. In contrast, absence of a jog is associated with abnormal, and unlinked, visceral *situs*. We also report the first mutation with organ-specific laterality defects, affecting heart alone.

### A framework for action of laterality genes

One interpretation for these data is that the embryo generates global lateralizing information, which it then transmits to the organs in a relatively all-or-none fashion, once a certain threshold is passed. This hypothesis, parts of which were originally promulgated by Wilhelmi (1921) and refined by Brown and Wolpert (1990) suggests that the embryo assembles a global asymmetry vector. If this information is not generated, no left–right information is available, which we believe would be manifest as Class 1 mutations: there is no inter-organ linkage and every embryo lacks a cardiac jog and manifests heterotaxy and even duplication of visceral organs. How, then, to explain Class 2 mutations, in which some embryos do manifest inter-organ linkage, either as *situs solitus* or *situs inversus*? We do not know, but speculate that such genes are needed to orientate the laterality vector. In other words, the vector does form, but its compass is not fixed in a uniform direction, and may vary, randomly, from embryo to embryo. We discuss this below.

### Class 1 genes: generation of a laterality 'vector'

Embryos with *ntl*, *flh* or *sur* mutations evidence no lateralization of *BMP4* in the heart. The heart does

Copyright © 2001 John Wiley & Sons, Ltd. 

not jog and the gut tends to remain midline. In addition, it has been noted that expressions of asymmetric genes, such as *lefty 1*, *lefty 2*, and *pitx2* are bilateral in the dorsal diencephalon, heart and gut in *ntl* and *flh* mutant embryos (Bisgrove *et al.*, 2000). We suggest that these mutations interfere with establishment of the global embryonic vector. Surprisingly, but concordant with the model, there is bilateral duplication of primordia of the two visceral organs studied, pancreas and liver. Because these are recessive mutations, presumably loss-of-function, this suggests the intriguing possibility that the left-sidedness of the liver is due normally to right-sided suppression. This might also be true for pancreatic rudiments, with left-sided suppression. Alternatively, it is possible that the pancreas, which normally forms as the fusion of dorsal and ventral buds, becomes bilateral in the mutant embryos because of mal-rotation of the gut and failure to fuse primordia. The mutations are present in 25% of progeny of heterozygous cross. Although there is some variability in phenotype, normal *situs* or *situs inversus* is essentially never present. This is compatible with a failure to generate lateralizing information or to transfer it to organs. Mutations in mice that might fall into this class would be those in Type IIB activin receptors (ActIIB). ActIIB –/– mutant embryos have severe *situs* abnormalities, including right lung isomerism, randomized cardiac position and asplenia (Oh and Li, 1997).

### Class 2 genes: vector orientation

In these mutations, the laterality vector is predicted to form in all mutant embryos, but may vary in orientation from embryo to embryo. Thus, in any one embryo it may be correctly orientated (producing *situs solitus*), reversed (producing *situs inversus*) or insufficient in net orientation away from the midline to provide lateralizing information (effectively absence of laterality, like Class 1).

What distinguishes this group from Class 1 is the coexistence of *situs inversus*, *situs solitus*, and heterotaxy in a single affected family. We examined cardiac *BMP4* in these mutants, to determine if the *BMP4* asymmetry moved around the heart tube as a direct read of the vector. It is left-dominant in *situs solitus* mutant, right-dominant in *situs inversus* mutants, and in heterotaxic mutants it is homogeneous or patchy (Chen *et al.*, 1997). In other words, if there is sufficient lateral polarizing 'force' to the vector, cardiac *BMP4* is a good gauge. If there is no vector, *BMP4* is not asymmetric at all.

Examples of known mouse genes which we believe fit in this class are *iv* and *inv*. About 50% of *iv* –/– embryos manifest normal *situs*, 50% *situs inversus*, and in some cases, heterotaxy is observed (Hummel and Chapman, 1959). Asymmetric expression pattern of laterality genes, such as nodal, is disrupted in *iv* mutant embryos. Normally, *nodal* is expressed in left lateral mesoderm (LPM). In *iv* mutant embryos, it can be detected in left LPM, or right LPM, or in both left and right LPM, or completely absent (Lowe *et al.*, 1996). *inv* mutant embryos tend more to have *situs inversus*, suggesting that the abnormality fixes the vector in a reversed direction (Yokoyama *et al.*, 1993). As would be predicted, *nodal* expression is only detected in right LPM in *inv* mutant embryos (Lowe *et al.*, 1996).

### Class 3 genes: organ-specific laterality

*laf* mutant embryos lack cardiac laterality (similar to the Class 1 effects upon the heart) but the viscera appear normal. Our interpretation is that there are components which differ among organs in how they interpret laterality information. In the mouse, *shh* and *lefty 1* mutations have profound influence on some but not all organs and may fit in this category (Meno *et al.*, 1998; Tsukui *et al.*, 1999).

### Implications for human defects

Several human pedigrees have been described with heritable laterality disorders. Interestingly, these pedigrees often include individuals with different manifestations of laterality disturbances. *Situs solitus*, *situs inversus*, heterotaxy and isomerism may coexist in a single affected family (Burn, 1991) (Kosaki and Casey, 1998). Although it is certainly conceivable that this variability in phenotype is due to incomplete penetrance, another explanation is that the mutation perturbs vector orientation, so that different individuals will have different phenotypes, like the Class 2 mutations. The severity of defects and organ disruption noted in all zebrafish embryos with Class 1 mutations would suggest that null mutants will be early embryonic lethal, so would come to light rarely, if ever, as obvious heritable patterns in humans.

Copyright © 2001 John Wiley & Sons, Ltd.

### Acknowledgements

Thanks to Jennifer Barrett for technical assistance. We appreciate the generosity of Drs Christiane Nüsslein-Volhard and Hans-Georg Frohnhöfer for providing mutant fish. This work is supported in part by NIH Grants RO1HL49579 and RR08888 to MCF. AMG is supported by a National Research Service Award (NIH, NHLBI, 1F32HL09585).

### References

Bisgrove BW, Essner JJ, Yost HJ. 2000. Multiple pathways in the midline regulate concordant brain, heart and gut left–right asymmetry. *Development* **127**: 3567–3579.

Brown NA, Wolpert L. 1990. The development of handedness in left/right asymmetry. *Development* **109**: 1–9.

Burn J. 1991. Disturbance of morphological laterality in humans. In *Biological Asymmetry and Handedness*, Bock GR, Marsh J (eds). Wiley: New York; 282–296.

Chen JN, Fishman MC. 1996. Zebrafish tinman homolog demarcates the heart field and initiates myocardial differentiation. *Development* **122**: 3809–3816.

Chen JN, van Eeden FJ, Warren KS, *et al.* 1997. Left–right pattern of cardiac BMP4 may drive asymmetry of the heart in zebrafish. *Development* **124**: 4373–4382.

Chin AJ, Tsang M, Weinberg ES. 2000. Heart and gut chiralities are controlled independently from initial heart position in the developing zebrafish. *Dev Biol* **227**: 403–421.

Danos MC, Yost HJ. 1995. Linkage of cardiac left–right asymmetry and dorsal–anterior development in *Xenopus*. *Development* **121**: 1467–174.

Danos MC, Yost HJ. 1996. Role of notochord in specification of cardiac left–right orientation in zebrafish and *Xenopus*. *Dev Biol* **177**: 96–103.

Hummel K, Chapman D. 1959. Visceral inversion and associated anomalies in the mouse. *J Hered* **50**: 9–13.

Kaufmann E, Knochel W. 1996. Five years on the wings of fork head. *Mech Dev* **57**: 3–20.

Kosaki K, Casey B. 1998. Genetics of human left–right axis malformations. *Semin Cell Dev Biol* **9**: 89–99.

Levin M, Johnson RL, Stern CD, Kuehn M, Tabin C. 1995. A molecular pathway determining left–right asymmetry in chick embryogenesis. *Cell* **82**: 803–814.

Levin M, Mercola M. 1998. Gap junctions are involved in the early generation of left–right asymmetry. *Dev Biol* **203**: 90–105.

Lowe LA, Supp DM, Sampath K, *et al.* 1996. Conserved left–right asymmetry of nodal expression and alterations in murine *situs inversus*. *Nature* **381**: 158–161.

Meno C, Shimono A, Saijoh Y, *et al.* 1998. Lefty-1 is required for left–right determination as a regulator of lefty-2 and nodal. *Cell* **94**: 287–297.

Milewski WM, Duguay SJ, Chan SJ, Steiner DF. 1998. Conservation of PDX-1 stucture, function, and expression in zebrafish. *Endocrinology* **139**: 1440–1449.

Mullins MC, Hammerschmidt M, Haffter P, Nusslein-Volhard C. 1994. Large-scale mutagenesis in the zebrafish: in search of genes controlling development in a vertebrate. *Curr Biol* **4**: 189–202.

Mullins MC, Hammerschmidt M, Kane DA, *et al.* 1996. Genes establishing dorsoventral pattern formation in the zebrafish embryos: the ventral specifiying genes. *Development* **123**: 81–93.

Odenthal J, Nüsslein-Volhard C. 1998. Fork head domain genes in zebrafish. *Dev Genes Evol* **208**: 245–258.

Oh SP, Li E. 1997. The signaling pathway mediated by the type IIB activin receptor controls axial patterning and lateral asymmetry in the mouse. *Genes Dev* **11**: 1812–1826.

Ramsdell AF, Yost HJ. 1998. Molecular mechanisms of vertebrate left–right development. *Trends Genet* **14**: 459–465.

Rodriguez Esteban C, Capdevila J, Economides AN, Pascual J, Ortiz A, Izpisua Belmonte JC. 1999. The novel Cer-like protein Caronte mediates the establishment of embryonic left–right asymmetry. *Nature* **401**: 243–251.

Schilling TR, Concordet J-P, Ingham PW. 1999. Regulation of left–right asymmetries in the zebrafish by *Shh* and *BMP4*. *Dev Biol* **210**: 277–287.

Supp DM, Witte DP, Potter SS, Brueckner M. 1997. Mutation of an axonemal dynein affects left–right asymmetry in *inversus viscerum* mice. *Nature* **389**: 963–966.

Tsukui T, Capdevila J, Tamura K, *et al.* 1999. Multiple left–right asymmetry defects in Shh(–/–) mutant mice unveil a convergence of the shh and retinoic acid pathways in the control of Lefty-1. *Proc Natl Acad Sci U S A* **96**: 11376–11381.

Wilhelmi H. 1921. Experimentelle Untersuchungen uber *Situs inversus* Viscerum. In *Archiv fur Entwicklungsmechanik der Organismen*, Roux W (ed.). Verlag Von Julius Springer: Berlin; 517–532.

Yokouchi Y, Vogan KJ, Pearse RV II, Tabin CJ. 1999. Antagonistic signaling by Caronte, a novel Cerberus-related gene, establishes left–right asymmetric gene expression. *Cell* **98**: 573–583.

Yokoyama T, Copeland NG, Jenkins NA, Montgomery CA, Elder FF, Overbeek PA. 1993. Reversal of left–right asymmetry: a *situs inversus* mutation. *Science* **260**: 679–682.

Copyright © 2001 John Wiley & Sons, Ltd.
